# Early administration of norepinephrine in sepsis: Multicenter randomized clinical trial (EA-NE-S-TUN) study protocol

**DOI:** 10.1371/journal.pone.0307407

**Published:** 2024-07-18

**Authors:** Ahlem Trifi, Sami Abdellatif, Asma Mehdi, Linda Messaoud, Eya Seghir, Nacef Mrad, Jalila Ben Khelil, Khaoula Ben Ismail, Takwa Merhaben, Hana Fradj, Amel Mokline, Amen Allah Messaadi, Hyem Khiari, Yasmin Garbaa, Nabiha Borsali Falfoul, Emna Ennouri, Radhouane Toumi, Mohamed Boussarsar, Oussama Jaoued, Souhail Atrous, Hassen Ben Ghezala, Nozha Brahmi, Insaf Trabelsi, Hatem Ghadhoune, Sabrine Bradaii, Mabrouk Bahloul, Rania Ammar, Fatma Medhioub Kaaniche

**Affiliations:** 1 Medical Intensive Care Unit (MICU), La Rabta Hospital, Tunis, Tunisia; 2 MICU, Abderrahmen Mami-hospital, Ariana, Tunisia; 3 MICU, Zaghouan Regional Hospital, Zaghouan, Tunisia; 4 Severe Burns Center, Ben Arous, Tunisia; 5 Department of Epidemiological Medicine and Statistics, Salah Azaiez Institute of Tunis, Tunis, Tunisia; 6 MICU, Habib Thameur-hospital, Tunis, Tunisia; 7 MICU, Farhat Hached-hospital, Sousse, Tunisia; 8 MICU,Tahar Sfar-hospital, Mahdia, Tunisia; 9 Urgent Medical Assistance Center, Tunis, Tunisia; 10 MICU, Habib Bougatfa-hospital, Bizerte, Tunisia; 11 MICU, Habib Bourguiba-hospital, Sfax, Tunisia; 12 MICU, Mahres Hospital, Sfax, Tunisia; CHU Nantes, FRANCE

## Abstract

One of the most important components of sepsis management is hemodynamic restoration. If the target mean arterial pressure (MAP) is not obtained, the first recommendation is for volume expansion, and the second is for norepinephrine (NE). We describe the methodology of a randomized multicenter trial aiming to assess the hypothesis that low-dose NE given early in adult patients with sepsis will provide better control of shock within 6 hours from therapy starting compared to standard care. This trial includes ICU septic patients in whom MAP decrease below 65 mmHg to be randomized into 2 groups: early NE-group versus standard care-group. The patient’s attending clinician will determine how much volume expansion is necessary to meet the target of a MAP > 65 mm Hg. If this target not achieved, after at least 30 ml/kg and guided by the available indices of fluid responsiveness, NE will be used in a usual way. The latter must follow a consensual schedule elaborated by the investigating centers. Parameters to be taken at inclusion and at H6 are: lactates, cardiac ultrasound parameters (stroke volume (SV), cardiac output (CO), E/E’ ratio), and P/F ratio. MAP and diuresis are recorded hourly. Our primary outcome is the shock control defined as a composite criterion (MAP > 65 mm Hg for 2 consecutive measurements and urinary output > 0.5 ml/kg/h for 2 consecutive hours) within 6 hours. Secondary outcomes: Decrease in serum lactate> 10% from baseline within 6 hours, the received fluid volume within 6 hours, variation of CO and E/E’, and 28 days-Mortality. The study is ongoing and aims to include at least 100 patients per arm. This study is likely to contribute to support the indication of early initiation of NE with the aim to restrict fluid intake in septic patients. **(ClinicalTrials.gov ID: NCT05836272).**

## Introduction

Sepsis is characterized by a systemic inflammation triggered with a severe infection and leading to inappropriate host response against infection. Typically, a vasoplegia with capillary leakage is noteworthy at the microcirculatory level [1]. The etiologic component of sepsis management involves the use of antibiotics and source elimination; symptomatic therapies include hemodynamic restoration and support for failing organs [2].

It is commonly recognized that hemodynamic restitution starts with volume expansion, when patient is thought to be afluid responder. Vasopressors, mainly norepinephrine (NE) as first-line therapy, should be used when mean arterial pressure (MAP) remains below the target after optimization of intravascular volume [2].

NE should be given as soon as sepsis resuscitation begins, according to a number of recent studies [3–5]. Indeed, when NE was administrated earlier than usually recommended, it improved MAP and cardiac output with a favorable effect on mortality [6]. At a median interval of 1.3 hours from ICU admission and exclusive administration of NE, MAP was adequately restored within a relatively short time (30 min) [7].

Furthermore, in comparable patients, it was linked to a greater survival than that predicted by severity scores [7]. In the same way, a retrospective study showed that the time to initiate NE was an independent factor of mortality [4]. In the subgroup that received early NE, the duration of hypotension and NE administration were shorter and the total dose of NE was lower than in the subgroup that received late NE [4]. On the other hand, giving lot fluids increases the risk of fluid overload, which worsens the prognosis for septic patients [8]. In the recent “CENSER” trial [5], the shock was controlled in 76%of patients in the early NE group versus 48% (p<0.001). In addition, the authors demonstrated that the incidences of cardiogenic pulmonary edema and recent arrhythmia were lower in the early NE group with respectively 22/155 (14.4%) vs 43/155 (27.7%), p = 0.004 and 17/155 (11%) vs 31/155 (20%), p = 0.03. In light of these arguments, it is tempting to limit fluid prescription at the early phase of hemodynamic control in septic states; by initiating NE early.

### Aim

We aim to assess the hypothesis that low-dose of NE given early, as soon as hypotension due to sepsis occur, and will provide better control of shock within 6 hours of treatment. This therapeutic regimen will be compared to standard care (including expansion with crystalloids, appropriate antibiotics, eradication of infection source and support of associated organ failure). Secondly, we seek to determine the effect of early NE on the administered fluid volume, cardiac output and the lactate level.

## Materials and methods

### Trial design

This is a multicenter parallel-group, randomized, interventional, active controlled trial. The 1:1 allocation ratio is regularly checked.

### Participants, eligibility criteria, and settings

The study is conducted in 11 Tunisian medical ICUs: Rabta, Ariana, severe burns department, Zaghouan, Habib Thameur Center, Mahdia, Farhat Hached Sousse Center, Sfax, Bizerte, Tunis Urgent Medical Assistance Center, and Mahres. The recruitment period for this study is scheduled to begin on September 15, 2023 and end over one year, i.e. September 15, 2024. Are eligible for our trial, all patients aging 18 or older, admitted in ICU, who present sepsis and a decrease in MAP below 65 mmHg. According to the sepsis 3 definitions update [1], sepsis is defined as an organ dysfunction caused by a documented or suspected infection. Organ dysfunction is represented by an increase in the Sequential [sepsis-related] Organ Failure Assessment (SOFA) score of 2 points or more. Exclusion criteria are: pregnancy, surgery indication, neoplasia, circumstances where fluid restriction is the rule (acute cardiogenic pulmonary edema…).

It should be noted that sepsis is only considered for inclusion when it occurred in the ICU to minimize the interventional bias. Septic patients who started receiving fluid therapy or vasopressive agents in their original departments (emergency or others) are excluded. Patients who die before primary outcome assessment are considered treatment failure.

In accordance with ethical principles of international guidelines including the Declaration of Helsinki, Council for International Organizations of Medical Sciences (CIOMS) International Ethical Guidelines, and International Conference on Harmonization (ICH) Good Clinical Practice (GCP) Guidelines, the protocol and other relevant documents are reviewed and approved by the institutional ethics Committee of the Northern Data Protection Committee of Tunisia on 11/04/2023 (CPP_37_2022_SI_noradrénaline) (supplement document). A written and informed consent is obtained from all patients or their legal representative. In case of urgent inclusion, we opt to call the legal parents to find out who can provide oral consent—or not; after explaining the study and its objectives. If oral consent is provided, the written consent will be completed the next day.

### Interventions

As soon as hypotension secondary to sepsis occur, the early NE arm receives the low-dose NE in addition to the standard therapeutic regimen that complies with the 2021 Surviving Sepsis Compaign (SSC) guidelines [2]. The low-dose NE is defined as a posology not strictly exceeding 0.25 mg per hour. The NE (Norepinephrine Bitartrate) is prepared as follows: 4 mg mixed with 250 ml of 5% glucose resulting in a final norepinephrine concentration of 0.016 mg/ml. For the placebo group, 250 ml of 5% glucose will be prepared. Both drugs will be infused via a peripheral line or a venous catheter. The intravenous infusion rate varies from 8 to 15 ml/hour, which is to be adjusted according to the body weight to obtain norepinephrine at 0.05 microgram/kg/min (ie 0.128 to 0.24 mg per hour) in continuous infusion. The placebo arm receives only the classic therapeutic regimen that complies with the 2021 SSC guidelines.

Standard Treatment regimen for sepsis includes expansion with crystalloid solution, appropriate antibiotics, control of the infection source, and support of associated organ failure (invasive ventilation, purification extra-renal…). The flow rate and volume of fluid expansion will be under the judgment of the clinician in charge and having for a hemodynamic objective a MAP > 65 mm Hg, in normotensive patients, and a higher threshold at 70–75 mm Hg in hypertensive patients. If this objective is not reached, after an optimal filling (at least 30 ml/kg), NE is authorized according to a usual schedule. Volume expansion is guided by the available indices of fluid responsiveness such passive leg raising (PLR), central venous pressure (CVP) variation, pulse pressure variation (PPV), ultrasound Inferior vena cava: (US-IVC) variation, etc…The common protocol for prescribing NE according to the usual schedule is as follows: norepinephrine by continuous intravenous injection, pure, and by electric syringe pump. The initial dosage is 1 mg/hour, the equivalent of 1 ml/hour. Then titrate according to the MAP to reach 65 mm Hg.

### Studied variables

Multiple and repeated measures are incorporated in the study design to ensure data integrity.

**Fig 1:**
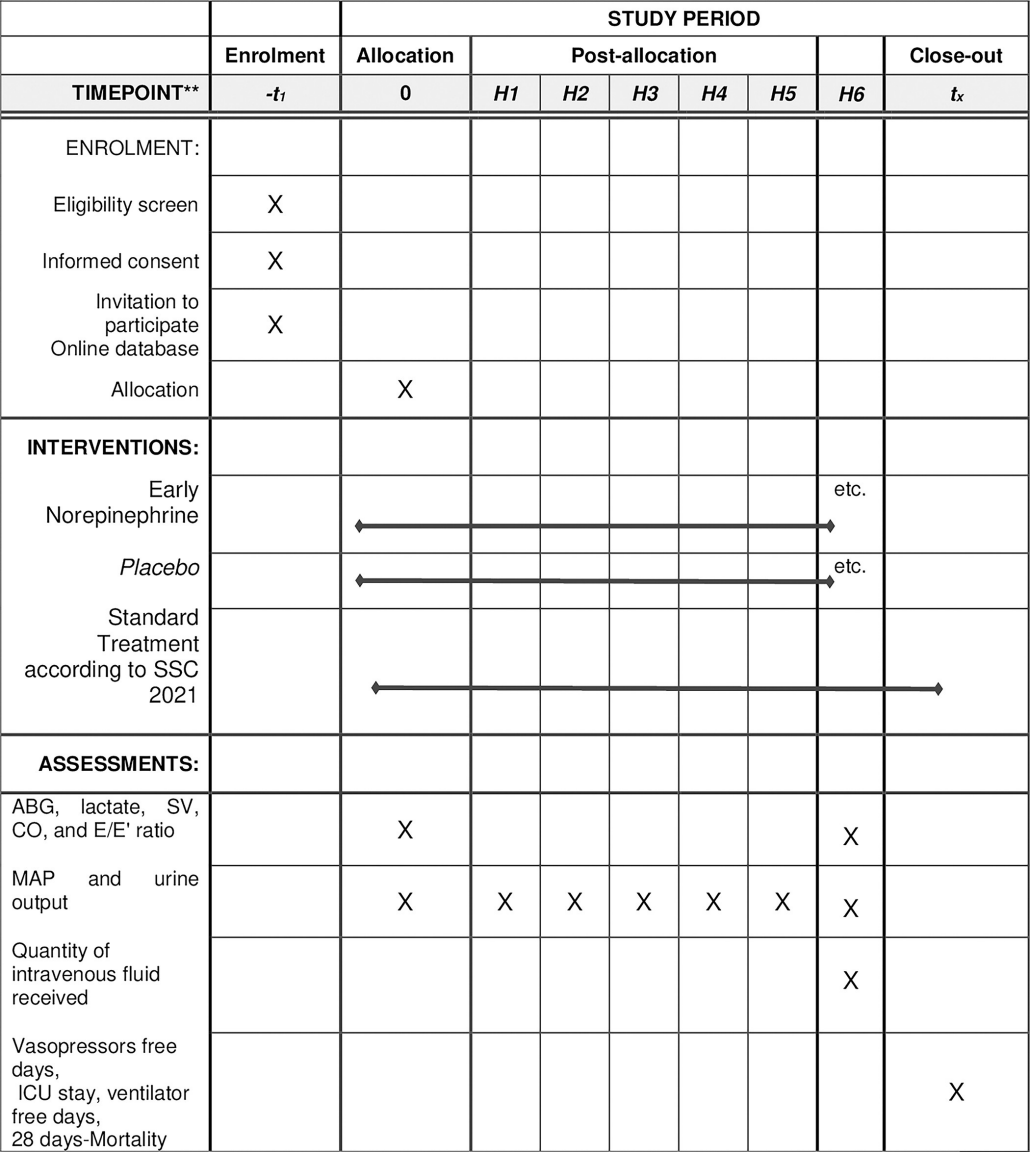
Spirit schedule of the study protocol. Once sepsis with hypotension is diagnosed in and consent is obtained, he is eligible and according to randomization he is allocated either to the early NE or placebo group. Are recorded clinical and biological data (blood gases, lactate, ionogram and renal function), basic cardiac echocardiography (stroke volume (SV), cardiac output (CO) and E/E’ ratio in addition to standard care according to SSC 2021, measurement of MAP every 15 mn and hourly monitoring of urine flow until H6. At H6: in addition to the clinical parameters of interest (MAP and diuresis), lactate levels, echo cardiographic parameters and fluid received. Clinical monitoring will be carried out until discharge in order to determine Vasopressors free days, ICU stay, ventilator free days, and 28 days-Mortality.

**At baseline:** clinical data related to the patient and septic state, hemodynamic status (in particular MAP, diuresis), biological data (blood gases with lactate), echocardiography parameters (stroke volume (SV), cardiac output (CO) and E/E’ ratio), hourly monitoring of MAP and urine output for 6 hours, hemodynamic objective achieved or not? and when? **At H6:** blood gases (P/F ratio and lactates) plus echocardiography parameters, quantity of fluids received over 6 hours. **Evolutionary parameters:** mortality, ventilation, hemodialysis, length of stay, etc.Note that all details of the EA-NE-S-TUN schedule of the study protocol are displayed in **Fig 1**.

### Outcomes (primary and secondary)

Our Primary Outcome is the shock control defined by a composite criterion (MAP > 65 mm Hg for 2 consecutive measurements AND urinary output > 0.5 ml/kg/h for 2 consecutive hours) within 6 hours. Secondary Outcomes are: Variation of CO (the 15% threshold is considered to define an increase in CO) within 6 hours, decrease in serum lactate > 10% from baseline within 6 hours, Quantity of intravenous fluid received, Improvement of SOFA at H24, vasopressors free days, ICU stay, ventilator free days, and 28 days-Mortality.

### Power and sample size

Herein, the major endpoint is the percentage of patients whose hypotension is restituted within 6 hours. According to the study by Permpikul C, et al (the CENSOR trial [5]), the shock control rate at 6 hours was significantly higher in the early norepinephrine group (76.1%) versus (48.4%) in the Placebo group. Consequently, the computation is performed as follows:

N per group = x

Where pA is the percentage of shock control within 6 hours in the early norepinephrine group (0.761) and pB is the percentage in the placebo group (0.484). Thus, for a targeted statistical power of at least 90% and at an alpha risk of 0.05, the size required for each arm is at least 96 patients. The desired effect size, based on the power and sample size mentioned above, was estimated at a gain of 1 hour 45 minutes from initial treatment to achieving target MAP and tissue perfusion goal.

### Interim analyses and stopping guidelines

Not applicable.

### Randomization (random number generation, allocation concealment, implementation)

Once included, patients are randomized according to a succession of six blocks of random permutations block 1: NE (norepinephrine)- P (placebo)-NE-P, block 2: P-NE-P-NE, block 3: NE-NE-P-P, block 4: P-P-NE-NE, block 5: P-NE-NE-P, block 6: NE-P-P-NE. Randomization is achieved via a computer-generated program, which also stratified patients based on age (≤ 55 years versus > 55 years), and SrOFA score (≤5 versus >5). The program of patient allocation is managed by a statistician blinded to the trial process.

### Blinding

The trial drug (early NE) is not disclosed to the patient, as per the single-blind protocol.

### Statistical analyses planned

Enrollment of at least 96 participants per group would provide a statistical power at 90% to assess the difference in the primary outcome between the two groups at a two-sided alpha error of 0.05. All primary and secondary outcomes analyses are based on the intention-to-treat principle. The primary outcome and other categorical variables are evaluated by the chi-square test or Fisher exact test, where appropriate. The independent link between early NE and shock resolution will be evaluated using proportion regression (Beta regression approach). Under violations of normality/Gaussian assumptions, we will employ the Wilcoxon-Mann-Whitney test to assess group differences for continuous variables. For the 28-day mortality analysis, time to death is calculated from date of sepsis diagnosis to date of death. Survival distributions in the two groups are estimated by Kaplan-Meier curves. Values of p less than 0.05 are considered to indicate statistical significance. All data analyses will be performed using SPSS Statistics version 20 (SPSS Inc.). Missing data will be handled using the imputation approach.

## Discussion

In this multicenter RCT, we plan to include at least 200 patients (n = 100 per group), which is, to our knowledge, the first and largest RCT involving almost all Tunisian ICUs reported to date. During the preparation of this manuscript, the methodology diffusion via several meetings between the investigators is taking place. We plan to close the study inclusion in September 2024.

The trial’s methodology requires a certain level of attention; particularly during the first six hours. Indeed, during this time interval, the patient is enrolled in his trial group, basic data (hemodynamic parameters, echocardiography and lactate level) are collected, quantity of volume expansion is determined (while being guided by responsiveness indices and monitored by hourly intakes of MAP, diuresis, heart rate, skin recoloration time, etc…), labeling a success or failure status according to the defined endpoint composite criterion. This entire assessment will be redone at H6 in order to assess the effect of the tested action (early NE or standard care) on the stroke volume, cardiac output, LV filling pressures and on the drop in lactate. The received fluids volume over the 6 hours and the total cumulative dose of NE administered during the septic episode will be also quantified.

Concerning the fluid balance, it is now well established that a positive balance is independently associated with an increased mortality rate in septic shock [9, 10]. It makes perfect sense to restrict fluid intake by initiating vasopressors early. In fact, the CENSER-RCT [6] found that the shock was more resolved by 6 hours in the early norepinephrine group (118/155 [76.1%] vs. 75/155 [48.4%]; *P* < 0.001); yet the 28-day mortality did not differ [5]. Despite the large sample size (n = 155 per arm), this trial only included one center and did not evaluate the immediate impact on stroke volume and CO. In the current trial, we try to circumvent these two limitations.

The EA-NE-TUN has several strengths. Aside from its multicenter-RCT design and rigorous methodology, no additional cost of care is added compared to standard care. Controlling for the correlation, introduced by the multicenter data design is necessary, even though this design significantly enhances the results’ external validity and applicability. The common shared database currently used and the stratified complex sampling strategy (SOFA, age, etc.) that we will use before statistical analysis could both help to rise statistical precision and control bias.

Other potential limitations that may be encountered during the study should be discussed. Firstly, the variability of echocardiography measures between centers and between practitioners from the same center. Secondly, during ICU guard, the resident may encounter concerns about patients’ inclusion. The latter is required to manage his duties while accurately ensuring the progress of study protocol, and carry out the echocardiography which is only possible under the supervision of a senior operator on site. Thirdly, lactate tests are not available 24/7 in some centers. To compensate such potential biases, we recommend that the entire procedure, both inclusion and the following 6 hours, be completed in the senior’s presence and during laboratory hours. A proportion of eligible patients may be excluded, constitutes the disadvantage of this recommendation.

Finally, given that the findings will only apply to this particular Tunisian community, we recommend additional research across different demographics and geographic areas to further validate the existing findings and ensure their generalizability.

## Conclusion

In conclusion, we presented the methodology of the EA-NE-TUN multicenter RCT, which is, to date, the first RCT involving the largest number of Tunisian medical ICUs. This study is likely to contribute to support the early initiation of norepinephrine and restrict fluid intake in septic patients.

## Supporting information

S1 Fig EA-NE-S-TUN spirit schedule of the study protocol. (TIF)

S1 File Ethical approval. (PDF)

S2 File SPIRIT check-list. (PDF)

S3 File Research protocol. (PDF)

S4 File Original ethical approval. (PDF)

S5 File Original research project. (PDF)

S6 File English version of research project. (PDF)

S1 Data (DOCX)

S2 Data (DOCX)

S3 Data (DOCX)

S4 Data (DOCX)

S5 Data (DOCX)
