# Supplementary material for: Early administration of norepinephrine in sepsis: Multicenter randomized clinical trial (EA-NE-S-TUN) study protocol
Source: PLoS One. 2024 Jul 18;19(7):e0307407. doi: 10.1371/journal.pone.0307407 (PMC11257256; doi:10.1371/journal.pone.0307407)
Supplement: S1 File — (PDF) [file pone.0307407.s002.pdf]

Tunisian republic
Ministry of Health

Personal Protection Committee

Tunis, April 06, 2023

Address:
Pasteur Institute of Tunis
13, Place Pasteur, B.P. 74
1002 Tunis, Belvedere
Tunisia

Opinion of the Committee for the Protection of Northerners

**Study title**	Early administration of norepinephrine in sepsis: Tunisian multicenter randomized clinical trial
**Unique National Registration Number**	TN2023-NAT-INS-99
**Number CPP-N**	CPPN_37_2022_SI_ Norepinephrine
**Abbreviated study title**	Early norepinephrine in sepsis
**Protocol code**	05-2022
**Promoter**	La Rabta Hospital
**Promoter's representative**	Pr Smai Mourali- Pr Sami Abdellatif
**Coordinating investigator**	Pr Ahlem Trifi
**Study product**	Norepinephrine-tartrate
**Indication**	Hypotension due to Sepsis
**Study phase**	IV
**Eudract number**	
**Subject of the submission**	Request for approval to carry out a therapeutic trial

Tunisian republic
Ministry of Health

Personal Protection Committee

The Personal Protection Committee met and reviewed the documents relating to this submission

List of members who voted:

Pr Lakhal Mohamed
Pr Hamdoun Moncef
Pr Boussema Fatma
Pr Slimane Hedia
Pr Ennigrou Samir
Pr Boubaker Samir
Dr Bouarrouj Samir
Mme Ben Ali Faten
Mme Lakhal Amel
Mme Masmoudi Ahlem

The members listed above are independent of the centers investigating this study.

This committee acts in accordance with good clinical practices and ICH recommendations.

The coordinating investigator Pr. Trifi Ahlem approached to carry out this research did not take part in the deliberations.

Decision of the Personal Protection Committee- North:

☒ Favorable ☐ Unfavorable ☐ Additional information

Date of signing: 11/04/2023

Vice-president Signature

Pr Hamdoun Moncef

Dr. Moncef HAMDOUN
Professeur à la Faculté de Médecine de Tunis
Chef de Service de Médecine Légale
Hôpital Charles Nicolle
