# Supplementary material for: Early administration of norepinephrine in sepsis: Multicenter randomized clinical trial (EA-NE-S-TUN) study protocol
Source: PLoS One. 2024 Jul 18;19(7):e0307407. doi: 10.1371/journal.pone.0307407 (PMC11257256; doi:10.1371/journal.pone.0307407)
Supplement: S2 File — (PDF) [file pone.0307407.s003.pdf]

SPIRIT check-list

- [1-5] **ADMINISTRATIVE INFORMATION**

1: TITLE : EARLY ADMINISTRATION OF NOREPINEPHRINE IN SEPSIS: TUNISIAN MULTICENTER RANDOMIZED CLINICAL TRIAL (EA-NE-S-TUN) STUDY PROTOCOL

2: TRIAL REGISTRATION : ClinicalTrials.gov ID: NCT05836272

3: PROTOCOL VERSION : original, **Issue Date**: septembre 20, 2023

4: FUNDING : This work is supported by the Tunisian intensive care association (Grant XX). The funders had no role in the design and conduct of the study; collection, management, analysis, or interpretation of the data; preparation, review, or approval of the manuscript; and decision to submit the manuscript for publication

5: ROLES AND RESPONSIBILITIES

5A: CONTRIBUTORSHIP : Conceptualization: AT, SA, JBK, NBF, NB, HG, Data curation: AM, LM, ES, NBM, YG, HBG, IT, Formal analysis: OJ, KBI, SA, TM, AMK, RA, Methodology: AT, EN, RT, HF, AMK, HK, FMK, SB, Writing –original draft: AT, SA, MB, Writing – review & editing: JBK, NBF, MB, AAM

5B: SPONSOR CONTACT INFORMATION : treasury of the Tunisian intensive care association (21698307750, email: atreanimation@gmail.com)

5C: SPONSOR AND FUNDER : Tunisian intensive care association

5D: COMMITTEES : not applicable

- [6-8] **INTRODUCTION**

6: BACKGROUND AND RATIONALE : Sepsis is characterized by a systemic inflammation triggered with a severe infection and leading to inappropriate host response against infection. At the microcirculatory level, a vasoplegia with capillary leakage is commonly notable [1]. Management of sepsis is based upon, the etiologic aspect which includes antimicrobials and eradication of the source, a symptomatic measures including hemodynamic restitution and assistance to failing organs [2].
It is now well known that hemodynamic restitution begins with volume expansion, followed by the use of vasopressors (mainly norepinephrine: NE as first-line therapy) when the mean arterial pressure target (MAP: reflecting the perfusion pressure organs) is not reached after optimizing the intravascular volume [2].
Recently, several studies have supported the benefit of administering NE at the start of resuscitation of sepsis [3-6]. Indeed, its administration at an earlier phase than usually recommended improved MAP and cardiac output with a favorable effect on mortality [7]. At a median interval of 1.3 hours from ICU admission and exclusive administration of NE, MAP was adequately restored within a relatively short time (30 min) and was associated with a better survival rate than that predicted by the severity scores of similar patients from other series reported in the literature [8]. A retrospective study showed that the time to initiate NE was an independent factor of mortality [9]. In the subgroup that received early NE, the duration of hypotension and NE administration was shorter and the total dose of NE was lower than in the subgroup that received late NE [9]. On the other hand, the administration of a large quantity of fluids inevitably increases the risk of fluid overload, which is a frequent complication in septic patients [10]. In the recent “CENSER” trial [6], the shock was controlled in 76% of patients in the early NE group versus 48% ($p<0.001$) and the incidences of cardiogenic pulmonary edema and recent arrhythmia were lower in the early NE group with respectively 22/155 (14.4%) vs 43/155 (27.7%), $p=0.004$ and 17/155 (11%) vs 31/155 (20%), $p=0.03$.
In front of all these arguments, it is therefore tempting to restrict fluid administration even to the initial phase of the hemodynamic management of sepsis by starting NE earlier.

7: OBJECTIVES : to assess the hypothesis that low-dose norepinephrine given early in adult septic patients with hypotension will provide better control of shock within 6 hours of treatment compared to standard care. Secondly, we study the effect of early NE on the administered fluid volume, cardiac output and the lactate level.

8: TRIAL DESIGN : randomized, single-blind, multicenter clinical trial

- [9-15] **METHODS: PARTICIPANTS, INTERVENTIONS, OUTCOMES**

  - 9: STUDY SETTING : medical ICUs in Tunisia
  - 10: ELIGIBILITY CRITERIA : all patients admitted in ICU aging 18 or older, giving (himself or legal representative) an informed consent in writing, in which sepsis is diagnosed according to the definitions updated by sepsis 3 consensus [1] and MAP decreased below 65 mmHg.
  - 11: INTERVENTIONS : The early NE arm receives the low-dose NE (4 mg mixed with 250 ml of 5% glucose) as soon as hypotension secondary to sepsis is observed in addition to the standard therapeutic regimen that complies with the 2021 Surviving Sepsis Compaign (SSC) guidelines.
  - 12: OUTCOMES : shock control defined by a composite criterion (MAP > 65 mm Hg for 2 consecutive measurements and urinary output > 0.5 ml/kg/h for 2 consecutive hours) within 6 hours.
  - 13: PARTICIPANT TIMELINE : 6 hours of sepsis onset

- 14: SAMPLE SIZE : at least 100 per group

- 15: RECRUITMENT : each participating center that has a patient who meets the inclusion criteria (sepsis with hypotension will incur the patient according to randomization

- [16-17] **METHODS: ASSIGNMENT OF INTERVENTIONS (FOR CONTROLLED TRIALS)**

  - 16: ALLOCATION : patients are randomized according to a succession of six blocks of random permutations block 1: NE (norepinephrine)- P (placebo)-NE-P, block 2: P-NE-P-NE, block 3: NE-NE-P-P , block 4: P-P-NE-NE, block 5: P-NE-NE-P, block 6: NE-P-P-NE. Randomization are performed using a computer-generated tool. Two groups are obtained: the NE group (early norepinephrine group) which receive NE at the beginning for the correction of hypotension and the Placebo group (standard treatment group).

  - 17: BLINDING (MASKING) : single-blind

- [18-20] **METHODS: DATA COLLECTION, MANAGEMENT, ANALYSIS**

  - 18: DATA COLLECTION METHODS : open data collection base between investigators is created and shared

19: DATA MANAGEMENT : regular periodical meetings are scheduled between investigators (who are only 2 in number by each center and designated beforehand) and this each time 20 patients are included. a verification of the taking of all the measures of interest will be carried out and ensure the correct approach of the trial protocol and the collection of data: the code identifying the patient according to the randomization and the key data (MAP, diuresis, hemodynamic objective achieved or not, echocardiographic parameters, lactates, etc.) must be entered and checked instantly as the test progresses. Other evolving data can be introduced later and verified during data management meetings.

20: STATISTICAL METHODS :
Outcomes: our Primary Outcome is the shock control defined by a composite criterion (MAP > 65 mm Hg for 2 consecutive measurements and urinary output > 0.5 ml/kg/h for 2 consecutive hours) within 6 hours. Secondary Outcomes are: Variation of CO (the 15% threshold is considered to define an increase in CO) within 6 hours, decrease in serum lactate > 10% from baseline within 6 hours, Quantity of intravenous fluid received, vasopressors free days, ICU stay, ventilator free days, 28 days-Mortality.
For a targeted statistical power of at least 90% and at an alpha risk of 0.05, the size required for each arm is at least 96 patients. All statistical analyzes will be performed by intention to treat and bilaterally.
Percentage of patients who reached the predefined hemodynamic objective and all the variables of interest will be compared between the 2 arms according to the appropriate statistical tests for independent samples. All statistical analyzes will be performed by intention to treat and bilaterally.

[21-23] **METHODS: MONITORING**

- **At baseline:** clinical data related to the patient and sepsis, hemodynamic status (in particular MAP, diuresis) biological data (blood gases with lactate), echocardiography parameters (stroke volume (SV), cardiac output (CO) and E/E' ratio), hourly monitoring of MAP and urine output for 6 hours, hemodynamic objective achieved or not? and when?
- **At H6:** blood gases (P/F ratio and lactates) plus echocardiography parameters, quantity of fluids received over 6 hours. **Evolutionary parameters:** mortality, ventilation, hemodialysis, length of stay, etc.)

[24-31] **ETHICS AND DISSEMINATION**

- Our clinical trial protocol respects the anonymity of collected data and complies with the ethical principles according to the Declaration of Helsinki. Patients or their legal representatives give their written consent to participate. A favorable opinion from the Northern Data Protection Committee of Tunisia on 11/04/2023 (CPP_37_2022_SI_noradrénaline) has been obtained (supplement document). The author(s) declare no competing interests with the study.
-

FIGURE (Figure 1. Spirit schedule of the study protocol)

REFERENCES:

1- Shankar-Hari M, Phillips GS, Levy ML, Seymour CW, Liu VX, Deutschman CS, et al. Developing a New Definition and Assessing New Clinical Criteria for Septic Shock: For the Third International Consensus Definitions for Sepsis and Septic Shock (Sepsis-3). JAMA 2016;315(8):775-87.

2- Evans L, Rhodes A, Alhazzani W, Antonelli M, Coopersmith CM, French C, et al. Surviving sepsis campaign: international guidelines for management of sepsis and septic shock 2021. Intensive Care Med. 2021;47(11):1181-247. doi:10.1007/s00134-021-06506-y

3- Hamzaoui O, Shi R. Early norepinephrine use in septic shock. J Thorac Dis. 2020;12(Suppl 1):S72-S77. doi: 10.21037/jtd.2019.12.50.

4- Bai X, Yu W, Ji W, Lin Z, Tan S, Duan K, et al. Early versus delayed administration of norepinephrine in patients with septic shock. Crit Care 2014;18:532.

5-Permpikul C, Tongyoo S, Viarasilpa T, Trainarongsakul T, Noppakaorattanamanee K. Early norepinephrine administration vs. standard treatment during severe sepsis/septic shock resuscitation: a randomized control trial. Intensive Care Med Exp 2017;5(Suppl. 2): 0426.
6-Permpikul C, Tongyoo S, Viarasilpa T, Trainarongsakul T, Chakorn T, Udompanturak S. Early Use of Norepinephrine in Septic Shock Resuscitation (CENSER). A Randomized Trial. Am J Respir Crit Care Med 2019;199:1097-105.

7-Hamzaoui O, Georger JF, Monnet X, Ksouri H, Maizel J, Richard C, et al. Early administration of norepinephrine increases cardiac preload and cardiac output in septic patients with life-threatening hypotension. Crit Care 2010;14:R142

8-Morimatsu H, Singh K, Uchino S, Bellomo R, Hart G. Early and exclusive use of norepinephrine in septic shock. Resuscitation 2004;62:249-54

9- Bai X, Yu W, Ji W, Lin Z, Tan S, Duan K, et al. Early versus delayed administration of norepinephrine in patients with septic shock. Crit Care 2014;18(5):532

10-Kelm DJ, Perrin JT, Cartin-Ceba R, Gajic O, Schenck L, Kennedy CC. Fluid overload in patients with severe sepsis and septic shock treated with early goal-directed therapy is associated with increased acute need for fluid-related medical interventions and hospital death. Shock 2015;43(1):68-73.

11. Sakr Y, Rubatto Birri PN, Kotfis K, Nanchal R, Shah B, Kluge S, et al. Intensive Care Over Nations Investigators. Higher Fluid Balance Increases the Risk of Death From Sepsis: Results From a Large International Audit. Crit Care Med. 2017;45(3):386-94.

12. Marik PE, Linde-Zwirble WT, Bittner EA, Sahatjian J, Hansell D. Fluid administration in severe sepsis and septic shock, patterns and outcomes: an analysis of a large national database. Intensive Care Med. 2017;43(5):625-32.
