# Supplementary material for: Early administration of norepinephrine in sepsis: Multicenter randomized clinical trial (EA-NE-S-TUN) study protocol
Source: PLoS One. 2024 Jul 18;19(7):e0307407. doi: 10.1371/journal.pone.0307407 (PMC11257256; doi:10.1371/journal.pone.0307407)
Supplement: S3 File — (PDF) [file pone.0307407.s004.pdf]

RESEARCH PROTOCOL
Date: 11/11/2022, VERSION: 0

TITLE: Early administration of norepinephrine in the management of severe sepsis (Randomized trial).

COORDINATING INVESTIGATOR: Pr Ahlem Trifi, Medical Intensive Care Unit, EPS la Rabta de Tunis

PROTOCOL:
Design: single-blind randomized clinical trial comparing 2 arms: a 1st arm receiving norepinephrine (NE) at low dose as soon as hypotension following sepsis is noted versus a 2nd which receives the placebo.

Inclusion criteria:
• Age 18 or older.
• The subject or their legal representative gives written informed consent.
• Diagnosis of sepsis with mean arterial pressure (MAP) < 65 mm Hg

Non-inclusion criteria:
• Diagnosis of septic shock before randomization (where NA requirements will be exceeded by the trial protocol)
• Pregnancy,
• Need for immediate surgical intervention,
• Advanced stage neoplasia
Exclusion criteria: Circumstances where water restriction is the rule:
• Acute pulmonary edema
• Acute coronary syndrome,

Randomization:
After inclusion, patients will be randomized according to a succession of six blocks of random permutations block 1: NA-P-NA-P, block 2: P-NA-P-NA, block 3: NA-NA-P-P , block 4: P-P-NA-NA, block 5: P-NA-NA-P, block 6: NA-P-P-NA. Randomization will be performed using a computer-generated tool. Two groups will be obtained: the NA group (early norepinephrine group) which will receive NA at the beginning for the correction of hypotension and Placebo group (standard treatment group).

Intervention:
The study molecule (norepinephrine) will be prepared using the approved and published protocol of Permpikul C, et al (cited in the research project) as follows: 4 mg mixed with 250 ml of 5% glucose resulting in a concentration final norepinephrine of 0.016 mg/ml (16 mg/L). For the placebo of the control group: 250 ml of 5% glucose will be prepared.

Both drugs will be infused via a peripheral line or venous catheter. The intravenous infusion rate varies from 8 to 15 ml/hour, adjusted according to body weight to obtain norepinephrine at 0.05 microgram/kg/min (i.e. 0.128 to 0.24 mg per hour) in continuous infusion.

All eligible patients will receive treatment for sepsis according to updated Surviving Sepsis Campaign guidelines. This will include expansion with crystalloid solution, appropriate antibiotics, control of the source of infection and support for associated organ failure (invasive ventilation, extra-renal purification, etc.).

The flow rate and volume of volume expansion will be under the judgment of the clinician in charge and having a hemodynamic objective of a MAP >65 mm Hg. If this objective is not achieved, after optimal filling (at least 30 ml/kg) and infusion of study drug (low-dose NE or placebo), vasopressors will be allowed according to a usual schedule.

Parameters of interest: hemodynamic: MAP, hourly diuresis, lactates, parameters obtained by echo cardiography (systolic ejection volume (SEV), cardiac output (CO), E/E' ratio to estimate the filling pressures of the left ventricle, and an oxygenation parameter (P/F ratio at baseline and at H6).

Calculation of sample (n)
Here, the judgment criterion is qualitative (percentage of shock correction within 6 hours). According to the study by Permpikul C, et al (the CENSOR trial [Ref 6]), the rate of shock control at 6 hours was significantly higher in the early norepinephrine group (76.1%) versus (48.4 %) in the Placebo group.

For a targeted statistical power of at least 90% and an alpha risk of 0.05, the necessary size for each arm is 100 patients.

All variables of interest will be compared between the 2 arms according to the statistical tests appropriate for independent samples.

All statistical analyzes will be carried out on an intention-to-treat and bilateral basis.

Dr Ahlem Trifi
Associated professor in Medical Resuscitation
Medical Intensive Care Department, La Rabta University Hospital
INVESTIGATOR COORDINATOR
