# Supplementary material for: Early administration of norepinephrine in sepsis: Multicenter randomized clinical trial (EA-NE-S-TUN) study protocol
Source: PLoS One. 2024 Jul 18;19(7):e0307407. doi: 10.1371/journal.pone.0307407 (PMC11257256; doi:10.1371/journal.pone.0307407)
Supplement: S4 File — (PDF) [file pone.0307407.s005.pdf]

Ministère de la santé
Hôpital La Rabta
Comité d'Ethique

Tunis, le 13/12/2022

A l'attention de Professeur Ahlem TRIFI

Objet : Avis éthique concernant votre travail de recherche

Le comité d'éthique, après étude du dossier dans le cadre du projet de recherche intitulé : «Administration précoce de la noradrénaline dans la prise en charge de l'état septique grave (Essai randomisé multicentrique)», donne un avis favorable.

Pr Mounir LAMLOUM

Président du Comité d'Ethique

الدكتور منير لملوم
أستاذ في الطب
مستشفى الرابطة تونس

Ministère de la santé
