# Supplementary material for: Early administration of norepinephrine in sepsis: Multicenter randomized clinical trial (EA-NE-S-TUN) study protocol
Source: PLoS One. 2024 Jul 18;19(7):e0307407. doi: 10.1371/journal.pone.0307407 (PMC11257256; doi:10.1371/journal.pone.0307407)
Supplement: S5 File — (PDF) [file pone.0307407.s006.pdf]

PROJET DE RECHERCHE

Date : 09/11/2022, VERSION : 1

A Mr le Président du comité d'éthique et de protection de personnes de Tunis

Objet : Demande d'approbation pour un essai thérapeutique

TITRE: Administration précoce de la noradrénaline dans la prise en charge de l'état septique grave (Essai randomisé).

INVESTIGATEUR CORDONNATEUR : Pr Ahlem Trifi (MCA en réanimation médicale, CHU la Rabta)

RATIONNEL : L'état septique grave (ESG) est caractérisé par l'inflammation systémique induite par une infection grave avec comme conséquence une réponse inappropriée de l'hôte contre cette infection. A l'échelle microcirculatoire on distingue une vasoplégie avec fuite capillaire [1]. La prise en charge de l'ESG comprend, outre le traitement spécifique qui inclut les antibiotiques et l'éradication de la source, une restauration des troubles hémodynamiques et une assistance des organes défaillants [2]. En général, la restauration des troubles hémodynamiques commence tout d'abord par l'expansion volémique, suivi par le recours aux vasopresseurs (principalement la noradrénaline en première intention) lorsque l'objectif de la pression artérielle moyenne (PAM : traduisant la pression de perfusion des organes) n'est pas atteint après avoir optimisé le volume intravasculaire [2].

Récemment, plusieurs études ont appuyé l'intérêt de l'administration de la noradrénaline (NA) tout au début de la réanimation d'un ESG [3-6]. En effet, son administration à une phase plus précoce qu'habituellement recommandée a amélioré la PAM et le débit cardiaque avec un effet favorable sur la mortalité [7]. A un intervalle médian de 1,3 heure de l'admission en réanimation et de l'administration exclusive de la noradrénaline, la PAM a été adéquatement restaurée dans un délai relativement court (30 min) et a été associée à un taux de survie meilleur que celui prédit par les scores de gravité de patients similaires d'autres séries rapportées dans la littérature [8]. Une étude rétrospective (213 patients en choc septique) a montré que le temps pour initier la NA était un facteur indépendant de surmortalité [9]. Dans le sous-groupe ayant reçu la NA précoce, la durée de l'hypotension et de l'administration de NA était plus courtes et la dose totale de NA était plus faible que dans le sous-groupe ayant reçu la NA tardive [9].

Le récent essai thaïlandais « CENSER » [6] a recruté 310 adultes diagnostiqués ayant un sepsis avec hypotension. Les patients ont été randomisés en deux groupes: NA précoce (n =155) et une thérapie standard (n=155). Le choc était contrôlé chez 76 % des patients du groupe NA précoce versus 48 % des patients du groupe contrôle (p<0,001) [6]. Il n'y avait aucune différence pour la mortalité. Les auteurs ont conclu que leurs découvertes confirment l'avantage l'administration précoce de la NA en cas de sepsis avec hypotension [6].

D'une autre part, l'administration d'une grande quantité de liquides augmente inévitablement le risque de surcharge liquidienne, qui est une complication fréquente chez les malades septiques [10]. Les mécanismes par lesquels l'excès d'administration de liquide peut aggraver le pronostic comprennent un œdème des tissus périphériques avec des risques de dysfonctionnement d'organe, un œdème pulmonaire avec risques d'hypoxémie et dégradation du glycocalyx endothélial avec des risques d'augmentation de la perméabilité vasculaire [11]. De plus, dans l'essai CENSER cité plus haut [6], les incidences de l'œdème pulmonaire cardiogénique et de l'arythmie récente étaient moindres chez le groupe NA précoce avec respectivement 22/155 (14,4 %) vs 43/155 (27,7 %), p=0,004 et 17/155 (11 %) vs 31/155 (20 %), p=0,03.

Face à tous ces arguments, il est donc tentant de restreindre l'administration de liquide même à la phase initiale de la gestion hémodynamique d'un ESG en démarrant la NA plus tôt.

Objectifs de l'étude :

Objectif général : Evaluer l'hypothèse que la noradrénaline à faible dose administrée précocement chez les patients adultes, atteints de sepsis avec hypotension, permettra un meilleur contrôle du choc dans les 6 heures du traitement par rapport aux soins standards.

Objectifs spécifiques :

- Etudier l'effet de l'adjonction précoce de la noradrénaline dans le sepsis avec hypotension sur la quantité de liquide administré, le débit cardiaque et sur le taux de lactates
- Comparer le risque de dysfonctions d'organes (évaluées par le recours à la ventilation mécanique et l'épuration extrarénale) et la mortalité entre le groupe recevant la noradrénaline précoce et celui qui recevra les soins standards.

Critères de jugement :

Principal : contrôle du choc défini par un critère composite (une PAM > 65 mm Hg pendant 2 mesures consécutives et débit urinaire > 0,5 ml/kg/h pendant 2 heures consécutives) et ceci dans les 6 heures de l'inclusion (cad lorsque le diagnostic de sepsis avec hypotension a été retenu). La PA doit être mesurée toutes les 15 mn après l'inclusion, soit par méthode automatisée non invasive ou via un cathéter artériel, si disponible.

Secondaires :

- Diminution du lactate sérique > 10 % par rapport à la valeur initiale
- Quantité de liquide reçue
- Recours à la Ventilation invasive
- Recours à l'épuration extrarénale

- Variation du débit cardiaque (DC) à H6 (le seuil de 15% est considéré pour définir une augmentation du DC).
- Variation du Ratio E/E’ (indice de surcharge échographique)
- Variation du rapport PaO2/FiO2 (paramètre d’oxygénation)
- Mortalité à 28 jours.

METHODOLOGIE :

Conception : essai clinique randomisé en simple aveugle comparant 2 bras : un 1er bras recevant la NA à faible dose dès la constatation de l’hypotension suivant un sepsis versus un 2ème qui reçoit le placebo. Les guidelines de SSC 2021 [2] seront suivis pour les 2 groupes. On prévoit de débuter l’essai en 15 septembre 2023 et le finir en 15 septembre 2024.

Critères d’inclusion :

- Âge de 18 ans ou plus.
- Le sujet ou son représentant légal donne son consentement éclairé par écrit.
- Diagnostic de sepsis selon les définitions actualisées par le consensus de sepsis 3 en 2016 [1]
- Pression artérielle moyenne < 65 mmHg

Critère de non inclusion:

- Diagnostic d’état de choc septique avant la randomisation (où les besoins de NA vont être dépassés le protocole de l’essai)
- Grossesse,
- Nécessité d'une intervention chirurgicale immédiate,
- Les néoplasies à un stade avancé

Critère d’exclusion: Les circonstances ou la restriction hydrique soit de règle :

- Œdème pulmonaire aigu
- Syndrome coronarien aigu,

Randomisation :

Après l'inclusion, les patients seront randomisés au hasard selon une succession de six blocs de permutations aléatoires **bloc 1 :** NA-P-NA-P, **bloc 2 :** P-NA-P-NA, **bloc 3 :** NA-NA-P-P, **bloc 4 :** P-P-NA-NA, **bloc 5 :** P-NA-NA-P, **bloc 6 :** NA-P-P-NA. La randomisation sera réalisée à l'aide d'un outil généré par ordinateur. Deux groupes seront obtenus : le groupe NA (groupe noradrénaline précoce) qui recevra la NA au début pour la correction de l’hypotension et groupe Placebo (groupe de traitement standard).

Intervention :
La molécule de l'étude (noradrénaline) sera préparée en empruntant le protocole approuvé et publié de Permpikul C, et al [6] comme suit : 4 mg mélangé avec 250 ml de glucosé à 5% aboutissant à une concentration de noradrénaline finale de 0,016 mg/ml (16 mg/L). Pour le placebo du groupe contrôle : 250 ml de glucosé à 5% sera préparé.
Les deux médicaments seront perfusés via une voie périphérique ou un cathéter veineux. Le débit de perfusion intraveineuse varie de 8 à 15 ml/heure, ajusté en fonction du poids corporel pour obtenir de la noradrénaline à 0,05 microgramme/kg/min (soit 0,128 à 0,24 mg par heure) en perfusion continue.
Tous les patients éligibles vont recevoir un traitement pour le sepsis selon le Surviving Sepsis Campaign : guidelines 2021 [2]. Cela comprendra l'expansion par soluté cristalloïde, antibiotiques appropriés, contrôle de la source de l'infection et support de défaillance d'organes associés (ventilation invasive, épuration extra rénale…).
Le débit et le volume de l'expansion volémique seront sous le jugement du clinicien en charge et ayant pour un objectif hémodynamique une PAM >65 mm Hg. Si cet objectif est non atteint, après un remplissage optimal (au moins 30 ml/kg) et perfusion du médicament de l'étude (NA à faible dose ou placebo), les vasopresseurs seront autorisés selon un schéma habituel.

Paramètres d'intérêt : d'ordre hémodynamique : PAM, diurèse horaire, lactates, paramètres obtenus par écho cardiographie (volume d'éjection systolique (VES), débit cardiaque (DC), ratio E/E' pour estimer les pressions de remplissage du ventricule gauche (PRVG)), pression veineuse centrale (PVC) et **un paramètre d'oxygénation** (ratio P/F à l'inclusion et à H6).
Tous ces paramètres vont être comparés entre les 2 bras selon les tests statistiques appropriés.

Calcul de l'échantillon (n)

Ici, le critère de jugement est qualitatif (pourcentage de correction du choc dans les 6 Heures). Selon l'étude de Permpikul C, et al [6], le taux de contrôle du choc à 6 heures était significativement plus élevé dans le groupe noradrénaline précoce (76,1 %) versus (48,4 %) dans le groupe Placebo.

$$N \text{ par groupe } = \frac{pA\,(1-pA) + pB\,(1-pB)}{(pB-pA)^2} \times (Z_{1-\alpha} + Z_{1-\beta})^2$$

Où pA est le pourcentage de contrôle du choc dans les 6 heures dans le groupe noradrénaline précoce (0,761) et pB est le pourcentage dans le groupe placebo (0,484).

Pour une puissance statistique visée d'au moins **90%** et à un risque alpha de **0,05**, la taille nécessaire pour chaque bras est au moins de **96 malades.**
Toutes les analyses statistiques seront réalisées en intention de traiter et en bilatéral.

<u>BIBLOGRAPHIE:</u>

1-Shankar-Hari M, Phillips GS, Levy ML, et al. Developing a New Definition and Assessing New Clinical Criteria for Septic Shock: For the Third International Consensus Definitions for Sepsis and Septic Shock (Sepsis-3). JAMA 2016;315:775-87.

2- Evans L, Rhodes A, Alhazzani W, et al. Surviving sepsis campaign: international guidelines for management of sepsis and septic shock 2021. *Intensive Care Med*. 2021;47(11):1181-1247. doi:10.1007/s00134-021-06506-y

3- O Hamzaoui, R Shi. *J Thorac Dis* 2020;12(Suppl 1):S72-S77 | http://dx.doi.org/10.21037/jtd.2019.12.50

4-Bai X, Yu W, Ji W, Lin Z, Tan S, Duan K, et al. Early versus delayed administration of norepinephrine in patients with septic shock. Crit Care 2014;18:532.

5-Permpikul C, Tongyoo S, Viarasilpa T, Trainarongsakul T, Noppakaorattanamanee K. Early norepinephrine administration vs. standard treatment during severe sepsis/septic shock resuscitation: a randomized control trial. Intensive Care Med Exp 2017;5(Suppl. 2): 0426.

6-Permpikul C, Tongyoo S, Viarasilpa T, et al. Early Use of Norepinephrine in Septic Shock Resuscitation (CENSER). A Randomized Trial. Am J Respir Crit Care Med 2019;199:1097-105.

7-Hamzaoui O, Georger JF, Monnet X, Ksouri H, Maizel J, Richard C, et al. Early administration of norepinephrine increases cardiac preload and cardiac output in septic patients with life-threatening hypotension. Crit Care 2010;14:R142

8-Morimatsu H, Singh K, Uchino S, et al. Early and exclusive use of norepinephrine in septic shock. Resuscitation 2004;62:249-54

9- Bai X, Yu W, Ji W, et al. Early versus delayed administration of norepinephrine in patients with septic shock. Crit Care 2014;18:532

10-Kelm DJ, Perrin JT, Cartin-Ceba R, et al. Fluid overload in patients with severe sepsis and septic shock treated with early goal-directed therapy is associated with increased acute need for fluid-related medical interventions and hospital death. Shock 2015;43:68-73.

11-Hippensteel JA, Uchimido R, Tyler PD, et al. Intravenous fluid resuscitation is associated with septic endothelial glycocalyx degradation. Crit Care. 2019;23:259
