# Supplementary material for: Early administration of norepinephrine in sepsis: Multicenter randomized clinical trial (EA-NE-S-TUN) study protocol
Source: PLoS One. 2024 Jul 18;19(7):e0307407. doi: 10.1371/journal.pone.0307407 (PMC11257256; doi:10.1371/journal.pone.0307407)
Supplement: S6 File — (PDF) [file pone.0307407.s007.pdf]

RESEARCH PROJECT

Date: 09/11/2022, VERSION: 1

To Mr. President of the Ethics and Personal Protection Committee of Tunis

Subject: Request for approval for a therapeutic trial

TITLE: Early administration of norepinephrine in the management of severe sepsis (Randomized trial).

INVESTIGATOR COORDINATOR: Professor Ahlem Trifi (associated professor, medical intensive care, Rabta University Hospital)

RATIONAL: Septic states (SS) are characterized by systemic inflammation induced by a severe infection with resulting to inappropriate host response to this infection. At the microcirculatory level, it is distinguished vasoplegia with capillary leak [1]. The management of SS includes, in addition to specific treatment based on antibiotics and eradication of infection source, restoration of hemodynamic disorders and assistance of failing organs [2]. In general, the restoration of hemodynamic disorders begins first with volume expansion, followed by the use of vasopressors (mainly norepinephrine as first line) when the goal of mean arterial pressure (MAP that reflect the perfusion pressure organs) is not achieved after optimizing the intravascular volume [2].

Recently, several studies have supported the benefit of administering norepinephrine (NE) at the start of SS resuscitation [3-6]. Indeed, its administration at an earlier phase than usually recommended improved MAP and cardiac output with a favorable effect on mortality [7]. At a median interval of 1.3 hours from admission to intensive care and exclusive administration of NE, MAP was adequately restored in a relatively short time (30 min) and was associated with a better survival rate than that predicted by severity scores of similar patients from other series reported in literature [8]. A retrospective study (213 patients with septic shock) showed that the time to initiate NE was an independent factor in excess mortality [9]. In the subgroup that received early NE, the duration of hypotension and NE administration were shorter and the total dose of NE was lower than in the subgroup that received late NE [9].

The Thai "CENSER" trial [6] recruited 310 adults diagnosed with sepsis with hypotension. Patients were randomized into two groups: early NE (n=155) and standard therapy (n=155). Shock was controlled in 76% of patients in the early NE group versus 48% of patients in the control group ($p<0.001$) [6]. There was no difference in mortality. The authors concluded that their findings confirm the benefit of early administration of NE in cases of sepsis with hypotension [6].

On the other hand, the administration of a large quantity of fluids inevitably increases the risk of fluid overload, which is a frequent complication in septic patients [10]. Mechanisms by which excess fluid administration may worsen prognosis include peripheral tissue edema with risks of organ dysfunction, pulmonary edema with risks of hypoxemia, and degradation of the endothelial glycocalyx with risks of increased vascular permeability [11]. Furthermore, in the CENSER trial cited above [6], the incidences of cardiogenic pulmonary edema and recent arrhythmia were lower in the early NE group with respectively 22/155 (14.4%) vs 43/ 155 (27.7%), p=0.004 and 17/155 (11%) vs 31/155 (20%), p=0.03.

In front of all these arguments, it is therefore tempting to restrict fluid administration even to the initial phase of hemodynamic management of an SS by starting NE earlier.

Study objectives:

General objective: To evaluate the hypothesis that low-dose norepinephrine administered early in adult patients suffering from sepsis with hypotension will allow better control of shock within 6 hours of treatment compared to standard care.

Specific objectives:

- Study the effect of early NE in sepsis with hypotension on the quantity of fluid administered, cardiac output and lactate levels

- Compare the risk of organ dysfunction (assessed by the use of mechanical ventilation and extrarenal purification) and mortality between the group receiving early NE and the group which will receive standard care.

Endpoints:

Main: control of shock defined by a composite criterion (a MAP > 65 mm Hg for 2 consecutive measurements and urinary flow > 0.5 ml/kg/h for 2 consecutive hours) and this within 6 hours of inclusion (i.e. when the diagnosis of sepsis with hypotension is made). Blood pressure should be taken every 15 min after inclusion, either by non-invasive automated method or via an arterial catheter, if available.

Secondary:

- Decrease in serum lactate > 10% compared to initial value
- Quantity of liquid received
- Use of invasive ventilation
- Use of renal replacement therapy (RRT)
- Variation in cardiac output (DC) at H6 (the threshold of 15% is considered to define an increase in DC).
- Variation in the E/E Ratio (ultrasound overload index)

- Variation of the PaO2/FiO2 ratio (oxygenation parameter)
- Mortality at 28 days.

METHODOLOGY:

Design: single-blind randomized clinical trial comparing 2 arms: a 1st arm receiving low-dose NE as soon as hypotension following sepsis is noted versus a 2nd arm receiving the placebo. The SSC 2021 guidelines [2] will be followed for both groups. The trial is planned to begin on September 15, 2023 and end on September 15, 2024.

Inclusion criteria:

- Age 18 or older
- Patients or their legal representative gives written informed consent.
- Diagnosis of sepsis according to the definitions updated by the Sepsis Consensus 3 in 2016 [1]
- Mean arterial pressure < 65 mmHg

Non-inclusion criteria:

- Diagnosis of septic shock before randomization (where NE requirements will be exceeded by the trial protocol)
- Pregnancy,
- Need for immediate surgical intervention,
- Advanced stage neoplasia

Exclusion criteria: Circumstances where water restriction is the rule:

- Acute pulmonary edema
- Acute coronary syndrome,

Randomization:

After inclusion, patients will be randomly randomized according to a succession of six blocks of random permutations block 1: NE-P-NE-P, block 2: P-NE-P-NE, block 3: NE-NE-P-P, block 4: P-P-NE-NE, block 5: P-NE-NE-P, block 6: NE-P-P-NE. Randomization will be performed using a computer-generated tool. Two groups will be obtained: the NE group (early NE group) which will receive NE at the beginning for the correction of hypotension and Placebo group (standard treatment group).

Intervention:

The study molecule (norepinephrine) will be prepared using the approved and published protocol of Permpikul C, et al [6] as follows: 4 mg mixed with 250 ml of 5% glucose resulting in a concentration of final norepinephrine of 0.016 mg/ml (16 mg/L). For the placebo of the control group: 250 ml of 5% glucose will be prepared.
Both drugs will be infused via a peripheral line or venous catheter. The intravenous infusion rate varies from 8 to 15 ml/hour, adjusted according to body weight to obtain

norepinephrine at 0.05 microgram/kg/min (i.e. 0.128 to 0.24 mg per hour) in continuous infusion.
All eligible patients will receive treatment for sepsis according to the Surviving Sepsis Campaign: guidelines 2021 [2]. This will include expansion with crystalloid solution, appropriate antibiotics, control of the source of infection and support for associated organ failure (invasive ventilation, RRT, etc.).
The flow rate and volume of volume expansion will be under the judgment of the clinician in charge with a hemodynamic objective of a MAP >65 mm Hg. If this objective is not achieved, after optimal filling (at least 30 ml/kg) and infusion of study drug (low-dose NE or placebo), vasopressors will be allowed according to a usual schedule.

Parameters of interest: hemodynamic: MAP, hourly diuresis, lactates, parameters obtained by trans-thoracic echocardiography (stroke volume (SV), cardiac output (CO), E/E' ratio to estimate the filling pressures of the left ventricle), and an oxygenation parameter (P/F ratio at baseline and at H6).
All these parameters will be compared between the 2 arms according to the appropriate statistical tests.

Calculation of sample (n):
Here, the main endpoint is qualitative (percentage of shock correction within 6 hours). According to the study by Permpikul C, et al [6], the rate of shock control at 6 hours was significantly higher in the early NE group (76.1%) versus (48.4 %) in the Placebo group.

$$\text{N per group} = \frac{pA\,(1-pA) + pB\,(1-pB)}{(pB-pA)^2} \times (Z_{1-\alpha} + Z_{1-\beta})^2$$

Where pA is the percentage of shock control within 6 hours in the early norepinephrine group (0.761) and pB is the percentage in the placebo group (0.484).
For a targeted statistical power of at least 90% and an alpha risk of 0.05, the necessary size for each arm is at least 96 patients.

All statistical analyzes will be carried out on an intention-to-treat and bilateral basis.

BIBLOGRAPHY:

1-Shankar-Hari M, Phillips GS, Levy ML, et al. Developing a New Definition and Assessing New Clinical Criteria for Septic Shock: For the Third International Consensus Definitions for Sepsis and Septic Shock (Sepsis-3). JAMA 2016;315:775-87.

2- Evans L, Rhodes A, Alhazzani W, et al. Surviving sepsis campaign: international guidelines for management of sepsis and septic shock 2021. *Intensive Care Med*. 2021;47(11):1181-1247. doi:10.1007/s00134-021-06506-y

3- O Hamzaoui, R Shi. *J Thorac Dis* 2020;12(Suppl 1):S72-S77 | http://dx.doi.org/10.21037/jtd.2019.12.50

4-Bai X, Yu W, Ji W, Lin Z, Tan S, Duan K, et al. Early versus delayed administration of norepinephrine in patients with septic shock. Crit Care 2014;18:532.

5-Permpikul C, Tongyoo S, Viarasilpa T, Trainarongsakul T, Noppakaorattanamanee K. Early norepinephrine administration vs. standard treatment during severe sepsis/septic shock resuscitation: a randomized control trial. Intensive Care Med Exp 2017;5(Suppl. 2):0426.

6-Permpikul C, Tongyoo S, Viarasilpa T, et al. Early Use of Norepinephrine in Septic Shock Resuscitation (CENSER). A Randomized Trial. Am J Respir Crit Care Med 2019;199:1097-105.

7-Hamzaoui O, Georger JF, Monnet X, Ksouri H, Maizel J, Richard C, et al. Early administration of norepinephrine increases cardiac preload and cardiac output in septic patients with life-threatening hypotension. Crit Care 2010;14:R142

8-Morimatsu H, Singh K, Uchino S, et al. Early and exclusive use of norepinephrine in septic shock. Resuscitation 2004;62:249-54

9- Bai X, Yu W, Ji W, et al. Early versus delayed administration of norepinephrine in patients with septic shock. Crit Care 2014;18:532

10-Kelm DJ, Perrin JT, Cartin-Ceba R, et al. Fluid overload in patients with severe sepsis and septic shock treated with early goal-directed therapy is associated with increased acute need for fluid-related medical interventions and hospital death. Shock 2015;43:68-73.

11-Hippensteel JA, Uchimido R, Tyler PD, et al. Intravenous fluid resuscitation is associated with septic endothelial glycocalyx degradation. Crit Care. 2019;23:259
