# Supplementary material for: Early administration of norepinephrine in sepsis: Multicenter randomized clinical trial (EA-NE-S-TUN) study protocol
Source: PLoS One. 2024 Jul 18;19(7):e0307407. doi: 10.1371/journal.pone.0307407 (PMC11257256; doi:10.1371/journal.pone.0307407)
Supplement: S5 Data — (DOCX) [file pone.0307407.s012.docx]

# Request for Change to Authorship

Check to confirm you have read [*PLOS ONE*’s authorship policy](http://journals.plos.org/plosone/s/authorship).

The authorship criteria for *PLOS ONE*, summarized below, are based on those outlined by the International Committee of Medical Journal Editors (ICMJE):

1. Conception and design of the work, acquisition of data, or analysis and interpretation of data
2. Drafting the article or revising it critically for important intellectual content
3. Final approval of the version to be published
4. Agreement to be accountable for all aspects of the work

Authors should meet all of the criteria; the contributions of all authors will be disclosed in the final publication. Any contributions that fall short of the criteria should be named in the Acknowledgments section of the manuscript. It is your responsibility to ensure that anyone named in the Acknowledgments consents to being named.

Check to confirm that all authors (including those to be added or removed) consent to the changes detailed below.

| **Reason for change in author list**  Please briefly describe the reason for adding/removing an author. | One author (Fatma Jarraya: FJ) was removed for withdrawal on her part. So, it was her colleague from the same service (Nacef Ben Mrad: NBM) who replaced her.  A statistician (Hyem Khiari: HK for her help to answer certain statistical analysis questions in this revision and she has undertaken to take care of the statistical analysis of the work once the data collection is finalized.  8 other authors were added, divided into 2 investigators per unit, because 4 medical ICUs expressed their willingness to join the study group after the first submission of this manuscript and began to include patients. |
| --- | --- |

# Final manuscript information

| **Manuscript number**  e.g., PONE-D-17-00000 | PONE-D-23-27726R1 - [EMID:c5d24df12cf6f2db] |
| --- | --- |
| **Complete author list, in correct order**  Please note any equal contributors with asterisks (*) or hashes (#) | Ahlem Trifi1¶*, Sami Abdellatif1¶, Asma Mehdi1&, Linda Messaoud1&, Eya Seghir1&, Nacef Ben Mrad2¶, Jalila Ben Khelil2&, Khaoula Ben Ismail3¶, Takwa Merhaben3&, Hana Fradj4¶, Amel Mokline4&, Amen Allah Messaadi4&, Hyem Khiari5&, Yasmin Garbaa6¶, Nabiha Borsali Falfoul6&, Emna Ennouri7¶, Radhouane Toumi7¶, Mohamed Boussarsar7& , Oussama Jaoued8¶, Souhail Atrous8, Hassen Ben Ghezala9¶, Nozha Brahmi9&, Insaf Trabelsi10¶, Hatem Ghadhoune10&, Sabrine Bradaii11¶, Mabrouk Bahloul11&, Rania Ammar12¶, Fatma Medhioub Kaaniche12& |
| [**Financial Disclosure**](http://journals.plos.org/plosone/s/disclosure-of-funding-sources)– including any additions/deletions necessary due to the change in authorship | no change |
| [**Competing Interests**](http://journals.plos.org/plosone/s/competing-interests)– including any additions/deletions necessary due to the change in authorship | no change |
| [**Acknowledgments statement**](http://journals.plos.org/plosone/s/submission-guidelines#loc-acknowledgments) Please acknowledge any removed authors if they contributed to the study in any way, as well as members of any author groups who do not meet our authorship criteria. | not applicable |

# Adding authors

## Individual author addition #1

| **Full name** | Rania Ammar |
| --- | --- |
| **Email address** | rania.ammarzayani@gmail.com |
| **Full affiliation** | Medical ICU, Mahres hospital, Sfax |

| This person contributed to **all** of the following:   1. Conception and design of the work, acquisition of data, or analysis and interpretation of data 2. Drafting the article or revising it critically for important intellectual content 3. Final approval of the version to be published 4. Agreement to be accountable for all aspects of the work |  |
| --- | --- |
| **Specific contributions:** | |
| Conceptualization |  |
| Data Curation |  |
| Formal Analysis |  |
| Funding Acquisition |  |
| Investigation |  |
| Methodology |  |
| Project Administration |  |
| Resources |  |
| Software |  |
| Supervision |  |
| Validation |  |
| Visualization |  |
| Writing – Original Draft Preparation |  |
| Writing – Review & Editing |  |

## Individual author addition #2 (if applicable)

| **Full name** | Fatma Medhioub Kaaniche |
| --- | --- |
| **Email address** | fatma_kaaniche@yahoo.fr |
| **Full affiliation** | Medical ICU, Mahres hospital, Sfax |

| This person contributed to **all** of the following:   1. Conception and design of the work, acquisition of data, or analysis and interpretation of data 2. Drafting the article or revising it critically for important intellectual content 3. Final approval of the version to be published 4. Agreement to be accountable for all aspects of the work |  |
| --- | --- |
| **Specific contributions:** | |
| Conceptualization |  |
| Data Curation |  |
| Formal Analysis |  |
| Funding Acquisition |  |
| Investigation |  |
| Methodology |  |
| Project Administration |  |
| Resources |  |
| Software |  |
| Supervision |  |
| Validation |  |
| Visualization |  |
| Writing – Original Draft Preparation |  |
| Writing – Review & Editing |  |

## Author group addition (if applicable)

| **Group or consortium name** |  |
| --- | --- |
| **Author who represents group** |  |

# Removing authors

## Author removal #1

| **Full name** |  |
| --- | --- |

| This person **did not** contribute to all of the following:   1. Conception and design of the work, acquisition of data, or analysis and interpretation of data 2. Drafting the article or revising it critically for important intellectual content 3. Final approval of the version to be published 4. Agreement to be accountable for all aspects of the work |  |
| --- | --- |
| This person consents to being acknowledged in the published paper. |  |

## Author removal #2 (if applicable)

| **Full name** |  |
| --- | --- |

| This person **did not** contribute to all of the following:   1. Conception and design of the work, acquisition of data, or analysis and interpretation of data 2. Drafting the article or revising it critically for important intellectual content 3. Final approval of the version to be published 4. Agreement to be accountable for all aspects of the work |  |
| --- | --- |
| This person consents to being acknowledged in the published paper. |  |
